# Navigating Uncertainty: Characterizing Rebound Lactic Acidosis in Pediatric Normothermic Machine Perfusion

**DOI:** 10.1111/petr.70500

**Published:** 2026-10-13

**Authors:** Chun‐Sing Huang, Alonzo Needum, Sarah G. Van Winkle, Gwendolyn B. Henry, Rayhan Neil Jhanji, Emma Hills, Naseem Ravanbakhsh, Abbas Rana, Kyle Soltys, George Mazariegos, N. Thao N. Galván, John Goss

**Affiliations:** ^1^ Division of Abdominal Transplantation, Michael E. DeBakey Department of Surgery Baylor College of Medicine Houston Texas USA; ^2^ School of Medicine Baylor College of Medicine Houston Texas USA; ^3^ Division of Hepatology Texas Children's Hospital, Baylor College of Medicine Houston Texas USA; ^4^ Division of Abdominal Transplantation, Children's Hospital of Pittsburgh University of Pittsburgh School of Medicine Pittsburgh Pennsylvania USA

**Keywords:** lactate acidosis, liver transplantation, normothermic machine perfusion, rebound lactic acidosis

## Abstract

**Background:**

Normothermic machine perfusion (NMP) is an emerging strategy for pediatric liver transplantation, but pediatric‐specific viability criteria remain undefined. Lactate clearance, extrapolated from adult protocols, is a central marker of allograft function. In our pediatric NMP (pNMP) experience, we observed a novel pattern: initial clearance followed by secondary rise, termed rebound lactic acidosis.

**Methods:**

We retrospectively reviewed pediatric liver allografts preserved on the TransMedics Organ Care System at two centers (January 2025 through June 2026). After excluding donors > 35 kg, 18 perfusions (17 transplants) were classified as rebound lactic acidosis (lactate rise > 0.5 mmol/L after initial clearance) or sustained clearance. Rebound cases were characterized by lactate kinetics, perfusion parameters, acid–base status, bile production, histology, and recipient outcomes.

**Results:**

Six allografts demonstrated rebound, with onset 1.0–6.6 h into perfusion. All sustained bile production throughout perfusion. Circuit alkalosis temporally correlated with rebound in three cases and improved with gas flow reduction. The first allograft was discarded; the remaining five were transplanted, including one perfused for 35.5 h. Recipients had elevated POD1 transaminases and INR that normalized by POD7; three are discharged home and two remain hospitalized, all with functioning allografts (follow‐up up to 312 days).

**Conclusions:**

Rebound lactic acidosis during pNMP may not indicate allograft nonviability and may be ameliorated by modifying circuit perfusion or pH. Lactate kinetics should be interpreted within a multiparametric viability framework incorporating perfusion adequacy, acid–base status, bile production, liver function, and histology. Pediatric‐specific criteria are needed to safely expand the donor pool.

## Graphical abstract

Normothermic machine perfusion in pediatric liver transplantation is a novel technique that may expand the donor pool. Here, authors describe a unique pattern of initial lactate clearance followed by non‐clearance, termed rebound lactic acidosis with early success in five pediatric transplants.

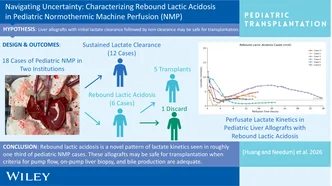

**Abbreviations**
ALT: alanine aminotransferase
AST: aspartate aminotransferase
DBD: donation after brain death
DCD: donation after circulatory death
HA: hepatic artery
INR: international normalized ratio
IRB: institutional review board
NMP: normothermic machine perfusion
OCS: Organ Care System
pNMP: pediatric normothermic machine perfusion
POD: postoperative day

## 1 Introduction

Pediatric liver transplantation is constrained by a persistent shortage of size‐matched donor allografts, contributing to waitlist mortality that disproportionately affects the youngest candidates [1, 2]. Normothermic machine perfusion (NMP) has emerged as a strategy to expand the usable donor pool and enable pretransplant functional assessment of allografts. Recent single‐center series have demonstrated the feasibility of pediatric NMP (pNMP) across small whole grafts, technical variant allografts, and donation‐after‐circulatory‐death (DCD) donors [3, 4, 5, 6]. However, standardized pediatric pretransplant viability criteria remain incompletely defined [7, 8].

Lactate clearance is widely used as a marker of graft metabolic function, and adult protocols commonly incorporate thresholds of 2.0 to 2.5 mmol/L within the early hours of perfusion [7, 8]. Emerging data, however, suggest that absolute thresholds may inadequately capture graft function, particularly in allografts with delayed clearance or dynamic lactate trajectories [2, 9]. Although variability in lactate kinetics during adult NMP is well documented, a secondary lactate rise following initial clearance—which we term “rebound lactic acidosis”—has not been systematically characterized in either adult or pediatric NMP [9, 10, 11].

In our two‐center pNMP experience, we observed six cases of rebound lactic acidosis. This phenomenon complicates pretransplant viability assessment because it is unclear whether the secondary lactate rise reflects intrinsic graft dysfunction or modifiable circuit‐related factors. Overly conservative interpretation risks non‐utilization of scarce pediatric grafts; overly permissive interpretation risks primary non‐function or early allograft dysfunction. Here, we describe our institutional experience with rebound lactic acidosis during pNMP and characterize the lactate kinetics, perfusion parameters, and postoperative outcomes of rebound and non‐rebound allografts.

## 2 Methods

### 2.1 Definition of Rebound Lactic Acidosis and Lactate Measurements

We defined rebound lactic acidosis as allografts that initially demonstrate lactate clearance, with persistent lactate reductions in the first 2 h of perfusion, that subsequently rise > 0.5 mmol/L. Lactate measurements were taken approximately every hour for the duration of perfusion for all cases. All lactate measurement data were provided by TransMedics and are shown in Figure 1 and Figure S1.

**FIGURE 1:**
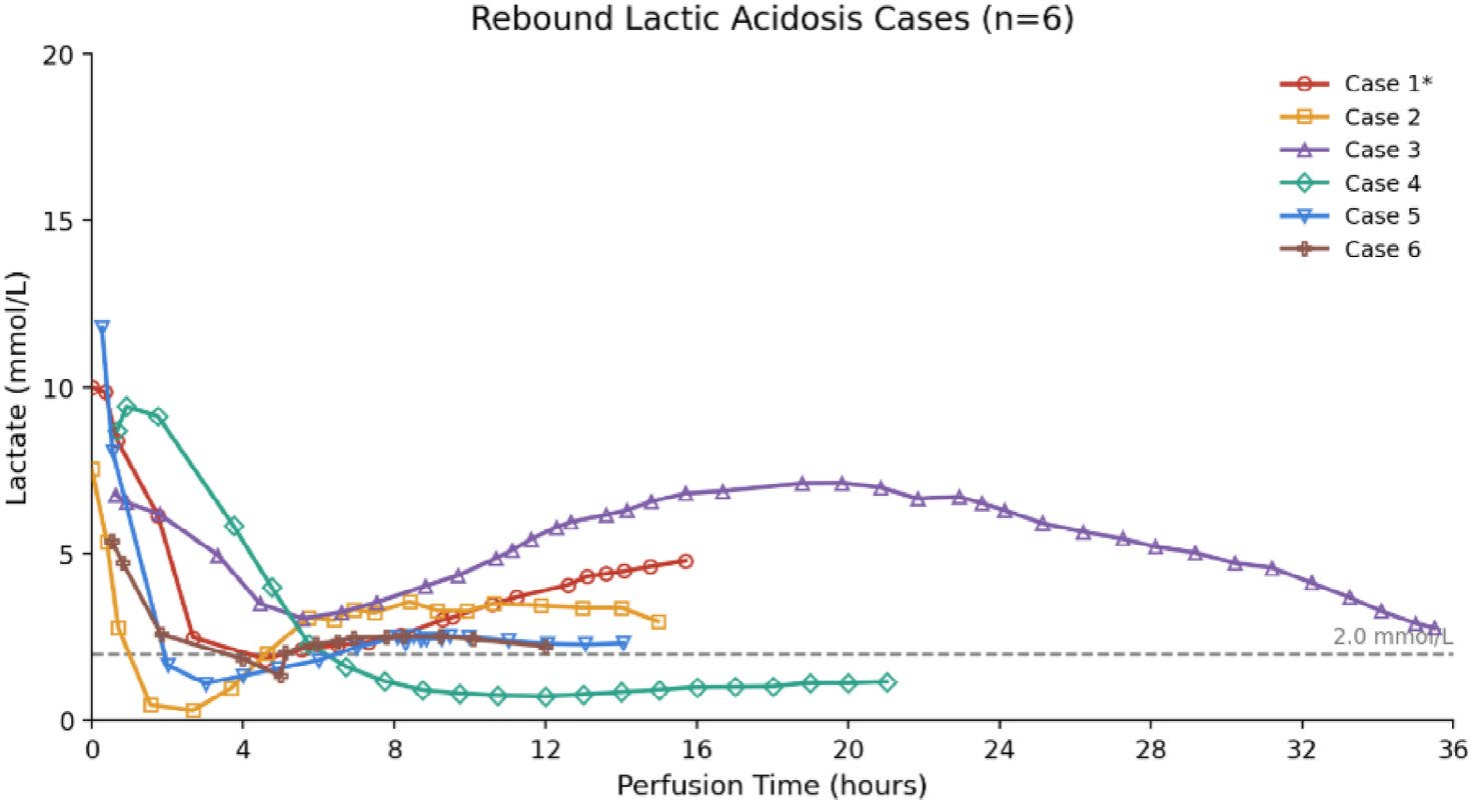
Perfusate lactate kinetics in pediatric liver allografts with rebound lactic acidosis during normothermic machine perfusion. Serial perfusate lactate is shown for all six grafts that developed rebound lactic acidosis (Cases 1–6). The dashed gray line indicates the institutional pre‐transplant viability threshold of 2.0 mmol/L. The asterisk (*) denotes the allograft that was not transplanted (Case 1); all other grafts were transplanted. Case 6 was referred from an outside institution.

### 2.2 Study Design and Population

We performed a multi‐institutional, retrospective review of pediatric normothermic machine perfusion (pNMP) cases utilizing the TransMedics Organ Care System (OCS) from January 2025 through June 2026 (*N* = 26). We excluded cases with donors > 35 kg (*N* = 9) to isolate analysis to small pediatric livers, as the OCS device requires technical adaptations to perfuse these allografts. This resulted in a final cohort of 18 cases: 6 with rebound lactic acidosis and 12 with sustained lactate clearance. Of the rebound lactic acidosis cases, 5 of the 6 allografts were used for transplantation. For each rebound case, the rates of lactate rise or clearance were calculated with linear regression. Linear interpolation was used to calculate the time of threshold crossing (2 mmol/L). Data were obtained from the institutional electronic health record. The study was reviewed and approved by the Baylor College of Medicine Institutional Review Board (Protocol H‐22045). IRB approval was obtained for University of Pittsburgh Medical Center (PRO15020384).

### 2.3 Pretransplant Viability Criteria and Perfusion Parameters

As NMP is a novel method of organ preservation for pediatric liver allografts, universally accepted pretransplant criteria have not yet been defined. Below are our institutional perfusion parameters:

- Total pump flow of 1.5–2 mL/min per gram of donor liver.
  ○ 1/3 of total flow directed to hepatic artery.
  ○ 2/3 of total flow directed to portal vein.
- Titrate hepatic artery mean pressure to 45 mmHg.
- Titrate mean portal venous pressure less than 10 mmHg.

Below are our current institutional criteria for pre‐transplant viability:

- Sustained lactate clearance or lactate < 2 mmol/L.
- Hepatic artery flow reaching 1/3 of total pump flow.
- Sustained bile production irrespective of total volume.
- Total perfusion time of at least 10 h.
- On‐pump liver biopsy if any of the above parameters are not met. Our criteria for acceptable biopsy results are no histological evidence of necrosis or ischemia.

### 2.4 Statistical Analysis

All analyses were performed in Microsoft Excel (Version 2603) and Python 3.14.3 with the NumPy, pandas, SciPy, and Matplotlib libraries. Continuous variables are reported as median (range).

## 3 Results

Six pNMP cases were identified with rebound lactic acidosis. Donor age ranged from 12 months to 7 years, and donor weight ranged from 8 to 34.5 kg (Table S1). Liver weight ranged from 280 to 700 g. Two of the six allografts were from DCD donors with warm ischemia time ranging from 11 to 28 min (one DCD case had 11 min of warm ischemia followed by 60 min of thoracoabdominal normothermic regional perfusion prior to procurement). All six allografts had normal hepatic arterial anatomy and did not require arterial reconstruction.

The time from cross‐clamp to machine perfusion ranged from 84 to 119 min. Hepatic artery flow ranged from 250 to 350 mL/min. All six allografts had initial clearance of lactate with ongoing bile production throughout machine perfusion. Timing of rebound lactic acidosis ranged from 60 to 395 min after the start of machine perfusion. The magnitude of lactate elevation ranged from 0.6 to 4.1 mmol/L (Figure 1, Cases 1–6). Three of the six cases had circuit pH > 7.5 that required alterations to gas flow to reduce alkalosis. Biopsy was obtained in three of six cases, all demonstrating normal architecture without evidence of necrosis. We obtained liver function tests of the circuit in two cases, with ALT of < 100 in both cases. In two of six cases, there was recovery of lactate clearance following rebound lactic acidosis. Following these pre‐transplant viability parameters, we discarded the first case with rebound lactic acidosis, and the five subsequent allografts were utilized for subsequent liver transplantation. Time on NMP ranged from 12.4 to 35.5 h.

Recipient age ranged from 4 months to 3 years old, and recipient weight ranged from 5 to 14 kg (Table S2). The UNOS allocation PELD score ranged from 2 to 31. Operation duration ranged from 307 min to 382 min with estimated blood loss ranging from 150 to 1000 mL. Post‐operative Day (POD) 1 transaminase ranged from an ALT of 430–1882. POD 7 ALT ranged from 48 to 299. Three of the five transplanted recipients are now discharged home with functional allografts; the remaining two remain hospitalized with functioning allografts. The duration of follow‐up extended up to 312 days.

To provide context for livers with rebound lactic acidosis with pNMP, we have included a reference cohort of 12 allografts utilized for liver transplant that had sustained lactate clearance (Table 1). Both sets of cases had similar donor size, hepatic artery flow, and time from cross‐clamp to on‐pump perfusion. However, the rebound cohort had a higher median recipient allocation PELD score (30 compared to 20 reference), higher median POD 1 transaminases and INR that were equalized by POD7 (POD1 ALT of 973 compared to 366 reference, POD 1 INR of 2.2 compared to 1.5 reference), and a longer median length of stay (30 days compared to 18 days reference).

**TABLE 1:** Characteristics of pediatric liver grafts with and without rebound lactic acidosis during normothermic machine perfusion.

Variable	Non‐rebound grafts (*n* = 12)	Rebound grafts (*n* = 6)
**Donor characteristics**		
Donor weight (kg)	14.0 (7.5–34.5)	12.3 (8.0–34.5)
Graft weight (g)	461 (322–918)	445 (280–700)
Warm ischemia time (min)^a^	27 (14–50)	18.5 (11–28)
Hepatic artery flow (mL/min)^b^	320 (150–400)	320 (250–350)
Cross‐clamp to pump time (min)	94 (80–190)	92 (84–119)
DCD donors, *n* (%)	8 (66.7)	2 (33.3)
**Perfusion characteristics**		
Time on pump (min)	922 (651–1489)	932.5 (746–2130)
Final perfusate lactate (mmol/L)	0.33 (0.30–3.31)	2.54 (1.31–4.79)
**Recipient characteristics** ^**c**^		
Recipient PELD score	20 (6–36)	30 (2–31)
Estimated blood loss (mL)	200 (100–1200)	250 (150–1000)
**Early post‐transplant outcomes** ^**c**^		
POD1 AST (U/L)^d^	946.5 (167–2560)	2068.5 (542–2846)
POD7 AST (U/L)	47.5 (26–329)	30 (18–141)
POD1 ALT (U/L)^d^	366 (164–1318)	973 (430–1882)
POD7 ALT (U/L)	120 (28–390)	132 (48–299)
POD1 INR	1.5 (1.2–4.0)	2.2 (1.5–6.7)
POD7 INR	1.2 (1.0–1.9)	1.1 (1.0–1.3)
**Clinical outcomes** ^**c**^		
Total length of stay (days)^e^	18 (8–54)	30 (7^+^–34)
ICU length of stay (days)^e^	11.5 (5–36)	10 (7^+^–27)
Total follow‐up (days)	245.5 (78–374)	61 (7–312)

*Note:* Continuous variables are presented as median (range); categorical variables are presented as *n* (%). All 12 non‐rebound grafts and 5 of the 6 rebound grafts were transplanted. Given the small number of rebound grafts, no formal statistical comparison between groups was performed and differences are presented descriptively.

Abbreviations: ALT, alanine aminotransferase; AST, aspartate aminotransferase; DCD, donation after circulatory death; ICU, intensive care unit; INR, international normalized ratio; PELD, Pediatric End‐Stage Liver Disease; POD, postoperative day.

^a^ Warm ischemia time was computed for DCD grafts only (non‐rebound *n* = 8; rebound *n* = 2).

^b^ Hepatic artery flow was not recorded for Case 6; the rebound column reflects *n* = 5.

^c^ Recipient, early post‐transplant, and clinical‐outcome rows reflect the five transplanted rebound grafts (Cases 2–6); Case 1 was discarded and is not included.

^d^ POD1 AST and ALT were not available for Case 6; the rebound column reflects *n* = 4 (Cases 2–5).

^e^ Superscript “+” denotes a censored value. Cases 5 and 6 remained hospitalized at the time of analysis, so their lengths of stay are minimum estimates.

## 4 Discussion

In this two‐center retrospective cohort study, six pediatric liver allografts demonstrated rebound lactic acidosis during NMP. All six allografts initially cleared lactate with persistent reduction in the first 2 h after initial perfusion but subsequently developed a secondary rise in lactate > 0.5 mmol/L while on pNMP. Although we discarded the first allograft with rebound lactic acidosis, the remaining five livers were transplanted. One allograft did not reach < 2 mmol/L of lactate anytime during perfusion. This liver was transplanted as on‐pump biopsies revealed no necrosis, and the liver had persistent lactate clearance for > 10 h following the initial rebound. The patient had mild reperfusion injury (POD1 INR of 2.4 and ALT of 584) that was resolved by POD7. With this preliminary data, our dual institutional outcomes are encouraging. Three of the five recipients have been discharged from the hospital with functional allografts; the remaining two are recovering with normal liver function in the hospital. Our preliminary data suggest that some elevations in lactate may represent a modifiable circuit parameter rather than intrinsic allograft dysfunction. In addition, the return of lactate clearance following rebound may not be necessary if biopsy and other perfusion parameters are adequate.

Lactate levels have been a central marker for pre‐transplant viability in NMP; however, prior studies have demonstrated significant variability in lactate clearance kinetics among NMP grafts. The OCS PROTECT trial demonstrated lactate clearance among successfully transplanted livers on NMP [10], but heterogeneity exists between the individual trajectories of these grafts. A 2024 multicenter cohort study of 509 livers undergoing NMP found that a subset of grafts took longer (4–6 h in this case) to achieve clearance. Recipients of this subset of livers followed a favorable clinical trajectory postoperatively, with 1‐year patient survival of up to 89%–100%. These results support the notion that allografts with delayed lactate clearance may remain transplantable [9].

Several graft‐intrinsic and circuit‐related factors may contribute to heterogeneity in lactate clearance kinetics for livers on NMP including steatosis, DCD donation, and circuit pH. None of the allografts in our cohort had liver biopsy‐evidence of hepatic steatosis, which is associated with primary nonfunction [12]. One study demonstrated allografts from DCD donors have increased rates of delayed or non‐clearance of lactate on NMP compared to brain dead donors [11]. However, Hofmann et al. and Clarke et al. both found no significant difference in metabolic function between DBD and DCD livers, measured via lactate clearance over time and cytochrome P450 1A2 activity, respectively [1, 9]. In our pediatric cohort, we had a total of 10 DCD allografts, two of which exhibited rebound lactic acidosis. It is unclear if DCD status contributed to their outcomes. We hypothesize another contributor of lactate non‐clearance is abnormal circuit pH. The gas flow in pNMP can reduce the PaCO2 levels significantly and circuit pH may exceed 7.6 [4]. We hypothesize that the liver cannot clear lactate when the circuit is significantly alkalotic. In three of our cases (Case 1, 2, and 5), alkalotic perfusion conditions appeared to temporally correlate with rising lactate levels. Our protocol is to minimize gas flow and occasionally pause gas flow if circuit pH exceeds 7.5.

This study has several limitations. We are limited to a small sample size from two transplant centers. The donor population is heterogenous, including variable size, age, and donor type. Although the transplanted recipients have functioning allografts, follow‐up data remain limited, extending up to 312 days across transplanted recipients. Long term outcomes such as ischemic cholangiopathy remain to be determined. Despite these limitations, this study is the first to characterize and demonstrate successful transplantation and function of pediatric liver allografts after rebound lactic acidosis. Overreliance on lactate alone may lead to non‐utilization of transplantable donor allografts.

Our current graft viability assessments and perfusion parameters have largely been extrapolated from adult NMP with a significant reliance on lactate. Our early experience suggests pNMP viability is more holistic and requires a multimodal assessment including perfusion criteria, acid–base status, bile production, and histological assessment along with lactate levels. Because the magnitude and clinical significance of rebound lactic acidosis in pNMP remain poorly characterized, adapting NMP to pediatric liver physiology is essential to safely expanding the donor pool with this technology.

## 5 Conclusion

Rebound lactic acidosis represents a previously uncharacterized pattern of lactate kinetics in pediatric normothermic machine perfusion, in which an initial clearance of lactate is complicated by a secondary lactate elevation. In this series, we discarded the first allograft with rebound, and the remaining five of six allografts were transplanted. All transplanted recipients have functioning allografts to date, with three discharged home and two remaining hospitalized. Taken together, these findings support a multifaceted approach to pretransplant viability in pNMP that integrates lactate kinetics with optimization of circuit parameters, acid–base status, bile production, and histology. Additional follow‐up data and multi‐center involvement will be needed to establish the safety of our technique and pediatric‐specific viability criteria that account for the unique metabolic and perfusion dynamics of small pediatric liver allografts.

## Conflicts of Interest

Authors C.H., T.G. and A.R. are paid by TransMedics to assist with organ recovery for other centers unrelated to this study.

## Supporting information


**Figure S1:** Perfusate lactate kinetics in pediatric liver allografts with sustained lactate clearance during normothermic machine perfusion. Serial perfusate lactate is shown for the 12 non‐rebound (sustained‐clearance) grafts. All 12 were transplanted.
**Table S1:** Donor and graft characteristics of the six pediatric liver allografts with rebound lactic acidosis.
**Table S2:** Recipient and early post‐transplant characteristics of the five transplanted pediatric liver allografts with rebound lactic acidosis.

## Data Availability

The de‐identified data that support the findings of this study are available from the corresponding author upon reasonable request and institutional approval. Patient‐level data are not publicly archived due to protections for pediatric human subjects.
