# Supplementary material for: Navigating Uncertainty: Characterizing Rebound Lactic Acidosis in Pediatric Normothermic Machine Perfusion
Source: Pediatr Transplant. 2026 Oct 13;30(10):e70500. doi: 10.1111/petr.70500 (PMC13662221; doi:10.1111/petr.70500)
Supplement: Supplementary file 1 [file PETR-30-e70500-s001.docx]

**Supplemental Figures**

**Supplemental Figure S1. Perfusate lactate kinetics in pediatric liver allografts with sustained lactate clearance during normothermic machine perfusion.** Serial perfusate lactate is shown for the 12 non-rebound (sustained-clearance) grafts. All 12 were transplanted.

**Supplemental Table S1. Donor and graft characteristics of the six pediatric liver allografts with rebound lactic acidosis.**

| **Case** | **Trans-planted** | **Donor age (months)** | **Donor weight (kg)** | **Graft weight (g)** | **Donor type** | **Cross-clamp to pump (min)** | **Warm ischemia time (min)** | **HA flow (mL/min)** | **PV flow (mL/min)** | **Bile output (mL)** | **Rebound onset (min)** | **Rebound magnitude (mmol/L)** | **Lactate recovery (min)** |
| --- | --- | --- | --- | --- | --- | --- | --- | --- | --- | --- | --- | --- | --- |
| 1 | No | 12 | 12.3 | 390 | DCD | 100 | 11 | 330 | 660 | 45 | 333 | 2.95 | Not recovered |
| 2 | Yes | 84 | 34.5 | 619 | DBD | 96 | — | 320 | 860 | 55 | 221 | 3.26 | 899 |
| 3 | Yes | 16 | 8.0 | 280 | DBD | 88 | — | 260 | 460 | 30 | 395 | 4.06 | 1448 |
| 4 | Yes | 24 | 13.5 | 500 | DCD | 119 | 28 | 350 | 800 | 75 | 60 | 0.60 | Not recovered |
| 5 | Yes | 24 | 12.0 | 700 | DBD | 85 | — | 250 | 800 | 10 | 180 | 1.42 | Not recovered |
| 6 | Yes | 17 | 12.2 | 350 | DBD | 84 | — | NR | NR | <25 | ~300^c^ | 1.19 | Not recovered |

Rebound onset denotes the interval from perfusion start to the onset of rising perfusate lactate after initial clearance; lactate recovery denotes the interval from perfusion start to renewed clearance, where this occurred. Rebound magnitude is the increase in perfusate lactate from nadir to peak. Em dashes (—) indicate that the variable was not applicable; NR indicates that the value was not recorded.

Abbreviations: DBD, donation after brain death; DCD, donation after circulatory death; HA, hepatic artery; NR, not recorded; PV, portal vein.

**Supplemental Table S2. Recipient and early post-transplant characteristics of the five transplanted pediatric liver allografts with rebound lactic acidosis.**

| **Case** | **Recipient age** | **Indication** | **Recipient weight (kg)** | **PELD score** | **POD1 AST/ALT (U/L)** | **POD7 AST/ALT (U/L)** | **POD30 AST/ALT (U/L)** | **POD1 INR** | **POD7 INR** | **POD30 INR** | **Total LOS (days)** | **ICU LOS (days)** | **Follow-up (days)** | **Complications** |
| --- | --- | --- | --- | --- | --- | --- | --- | --- | --- | --- | --- | --- | --- | --- |
| 2 | 10 months | Biliary atresia | 10 | 30 | 542/430 | 18/48 | 21/15 | 1.5 | 1.0 | 1.0 | 31 | 27 | 312 | None |
| 3 | 4 months | OTC deficiency | 5 | 31 | 1920/584 | 24/58 | 34/28 | 2.6 | 1.1 | NR | 34 | 19 | 72 | None |
| 4 | 5 months | Biliary atresia | 7 | 17 | 2846/1882 | 30/158 | 32/26 | 2.2 | 1.1 | NR | 19 | 10 | 61 | None |
| 5 | 3 years | Re-transplant (HAT), biliary atresia | 14 | 31 | 2217/1362 | 141/299 | NR | 2.1 | 1.3 | NR | 7⁺ | 7⁺ | 7 | None |
| 6^b^ | 10 months | Hepatoblastoma | 6.3 | 2 | NR | 95/132 | 28/34 | 6.7 | 1.2 | 1.0 | 30⁺ | 10 | 30 | None |

Cases 2–6 are shown; Case 1 was discarded and is not included. NR indicates that the value was not recorded. ⁺ denotes a censored value: Cases 5 and 6 remained hospitalized at the time of analysis, so their lengths of stay are minimum estimates.

Abbreviations: ALT, alanine aminotransferase; AST, aspartate aminotransferase; HAT, hepatic artery thrombosis; ICU, intensive care unit; INR, international normalized ratio; LOS, length of stay; NR, not recorded; OTC, ornithine transcarbamylase; PELD, Pediatric End-Stage Liver Disease; POD, postoperative day.
